# Supplementary material for: Theoretical Explanation for the Rarity of Antibody-Dependent Enhancement of Infection (ADE) in COVID-19
Source: Int J Mol Sci. 2022 Sep 26;23(19):11364. doi: 10.3390/ijms231911364 (PMC9569501; doi:10.3390/ijms231911364)
Supplement: Supplementary file 1 [file ijms-23-11364-s001.zip › ijms-1926403-supplementary.pdf]

Table of contents:

1. Supporting Tables 2
2. Supporting Figures 7
3. Supporting Methods for model description and parameters estimation 11
   S1. Parameters for antibody-response generation module (“Model-Ab”) 11
   S2. Parameters for “Complete model” 11
   S3. Reduced equation system of viral propagation in culture of HAE cells 11
   S4. Modified system of equations, including interaction with pre-existing antibodies 11
4. Supporting Abbreviations 12
5. Supporting References 12

1. Supporting Tables

Table S1. Model variables.

Variable	Compartment	Definition	Module	Initial value (per ml)
F_{RNA}	Lungs	RNA number in saliva	"Model-Ab"	188257
V_f	Lungs	Concentration of free viral epitopes	"Model-Ab"	10^8
Va	Lungs	Concentration of viral epitope-antibody complexes	"Model-Ab", Complete model	0
Ap	Lungs	Number of neutral lungs antigen-presenting sites	"Model-Ab", Complete model	$1 \cdot 10^5$
Aps_{lung}	Lungs	Number of activated lungs antigen-presenting sites	"Model-Ab", "Complete model"	0
Aps_{lo}	Lymphoid organs	Number of activated lymphoid organs antigen-presenting sites	"Model-Ab", "Complete model"	0
B	Lymphoid organs	Number of naïve B-cells	"Model-Ab", "Complete model"	10000
Pl	Lymphoid organs	Number of IgG-producing plasma cells	"Model-Ab", "Complete model"	0
B_m	Lymphoid organs	Number of memory B-cells	"Model-Ab", "Complete model"	0
A_{lo}	Lymphoid organs	Concentration of antibodies in body fluids and lymphoid organs	"Model-Ab", "Complete model"	0
A_{lung}	Lungs	Concentration of antibodies in lungs	"Model-Ab", "Complete model"	0
Pl_{mod}	Lung	Plasma cells	"Model-Cv"	$2 \cdot 10^6$
Virus	Lungs	Concentration of viral particles in lungs	"Model-Cv"	10^5-$5 \cdot 10^7$ (mean $2.67 \cdot 10^7$)
M_A	Lungs	Number of healthy macrophages	"Model-Cv", "Complete model"	200000
M^*	Lungs	Number of infected macrophages	"Model-Cv", "Complete model"	0
P_n	Lungs	Number of healthy pneumocytes	"Model-Cv", "Complete model"	$1.8 \cdot 10^7$
P_i	Lungs	Number of infected pneumocytes	"Model-Cv", "Complete model"	0
V	Lungs	Concentration of free viral epitopes	"Complete model"	$6.67 \cdot 10^8$

Table S2. Parameter values for antibody-generation module ("Model-Ab")

Parameter	Definition	Value	Ref.
A_{RNA}	Parameter of RNA kinetic approximation	798292 RNA number	Estimated from [1]

x_0	Parameter of RNA kinetic approximation	2.998 days	Estimated from [1]
w_1	Parameter of RNA kinetic approximation	2.069 days	Estimated from [1]
w_2	Parameter of RNA kinetic approximation	2.069 days	Estimated from [1]
w_3	Parameter of RNA kinetic approximation	1-4 days (mean 2.378)	Estimated from [1]
β_{RNA}	Constant of proportionality between number of SARS-CoV 2 RNA number and amount of free viral epitopes	200-1000 V/RNA number (mean 532)	Estimated from [1]–[3]
k_{v1}	Virus-antibody association rate	$k_{v-1}/(K_d \cdot 6 \cdot 10^{20})=4.3 \cdot 10^{-12}$ V·days$^{-1.}$	[4], [5]
k_{v-1}	Virus-antibody dissociation rate	25.8 days^{-1}	[4], [5]
K_d	Virus-antibody dissociation constant	$1 \cdot 10^{-8}$ M	[4]
C_{v1}	Maximal rate of viral protein-antibody complexes	10^{11} day^{-1}	Estimated from [6], [7]
C_{v2}	Viral protein-antibody complexes half-decay constant	$5.3 \cdot 10^{12}$ V	Estimated from [1], [6], [7]
χ_{ap}	Neutral APSs generation and degradation in the absence of virus (homeostasis rate of neutral APSs)	0.1155 day^{-1}	[8]–[10]
β_{apm}	Rate of lung APSs activation	1.432 day^{-1}	Estimated from [6]
ϕ	Half saturation constant of APS activation	10^{10} V	Estimated from [11], [12]
δ_{ap}	Rate of APS_{lung} deactivation	0.3 day^{-1}	Estimated from [6]
k_{s1}	The rate of APSs transition from lungs to lymphoid organs	0.4 day^{-1}	-
k_{s-1}	The rate of APSs transition from lymphoid organs to lungs	0.1 (cells·day)$^{-1}$	-
v	Conversion coefficient reflecting lymphoid organs:lung volume ratio	0.4	Estimated from [11], [13]
χ_b	B-cells generation and degradation in the absence of virus (homeostasis rate of B cells)	358 day^{-1}	[6]
π_b	Naïve B-cells proliferation rate	0.00177 (APS·day)$^{-1}$	[14]
π_{diff}	The rate of differentiation	$1 \cdot 10^{-5}$-$2 \cdot 10^{-4}$ APS·day^{-1} (mean 10^{-4})	donor-specific; estimated from [1]
π_{pl}	Differentiation of naïve B-cells in long-lived plasma cells	$\pi_{diff}=10^{-4}$ (APS·day)$^{-1}$	donor-specific; estimated from [1]
π_{bm}	Differentiation of naïve B-cells in memory B-cells	$\pi_{diff}=10^{-4}$ (APS·day)$^{-1}$	donor-specific; estimated from [1]
π_{ps}	Differentiation of naïve B-cells in short-lived plasma cells	$2 \cdot \pi_{diff}=2 \cdot 10\text{-}^{4}$ (APS·day)$^{-1}$	donor-specific; estimated from [1]
π_{ml}	Differentiation of memory B-cells in long-lived plasma cells	$\pi_{diff}/10=10^{-5}$ (APS·day)$^{-1}$	Estimated from [1]
t_{naive}	Lag-time between the appearance of APSs (at t_0) and initiation of naive B-cells differentiation and proliferation	6-12 days (10.5 day)	donor-specific; estimated from [1], [15], [16]
t_{memory}	Lag-time between the appearance of APSs (at t_0) and initiation of memory B-cells differentiation and proliferation	3-5 days (mean 4 day)	donor-specific; estimated from [15], [16]
δ_{pl}	Rate of plasma cells degradation	10^{-4}-1 days^{-1} (mean 0.165)	donor-specific; estimated

			from [1]
k_{bm1}	*Bm* growth rate	10^{-5} day^{-1}	Estimated from [14]
k_{bm2}	Lymphoid organs maximum capacity	5000 cells	Estimated from [11], [12]
α_{pl}	Rate of IgG generation by long-living plasma cells	10^{9} number·(Pl·day)$^{-1}$	[17]–[19]
δ_a	The natural decay rate of the A_b	0.04 day^{-1}	[6], [14], [20]
ka_1	The rate of antibodies transition from lungs to lymphoid organs	0.95 day^{-1}	-
ka_{-1}	The rate of antibodies transition from lymphoid organs to lungs	1 day^{-1}	-
ω	Background level of IgGs in serum	1-2 (mean 1.01)	donor specific;<br>estimated from [1]
Ig_0	Scaling coefficient that depended on the type of antibody test	$1{\cdot}10^{13}$ Numbers	Estimated from [1], [11]

Table S3. Parameter values for the virus replication module (“Model Cv”)

Parameter	**Definition**	**Value**	**Ref.**
k_B	Plasma cells production rate	40	[21]
μ_B	Plasma cells degradation rate	0.02 day^{-1}	[21]
α_B	Rate of plasma cells proliferation upon contact with viral particles	10^{-14} (Virus·day)$^{-1}$	[21]
χ_m	Macrophages generation and degradation in the absence of virus (homeostasis rate of alveolar macrophages)	0.1 $days^{-1}$	[8]–[10]
α_c	Maximum rate constant for the antibody–virus complex formation and subsequent engulfment	10^{-12}-10^{-8} $(cells^2 day)^{-1}$ (mean $6{\cdot}10^{-10}$)	Estimated from [7]
ρ	Probability of macrophages to become infected	10^{-5}-10^{-1}	[21]
σ	Rate of infected macrophages recovery	0.2 day^{-1}	[21]
μ	Rate of degradation of infected macrophages	2 day^{-1}	[22], [23]
p	Rate of virus production by pneumocytes	6665 Virus/(cells·day)	Estimated from [24]
α_v	Rate of viral phagocytosis by the macrophages	$8{\cdot}10^{-9}$ (cells·day)$^{-1}$	[20], [25]
δ_v	Rate of viral degradation	50-300 day^{-1} (mean 207.6)	[21]
β_{inf}	Rate of pneumocytes infection	10^{-7}-$5{\cdot}10^{-5}$ (Virus·day)$^{-1}$ (mean $4.72{\cdot}10^{-6}$)	Estimated from [7]
δ_p	Death rate of infected pneumocytes	2.3 day^{-1}	Estimated from [24]
α_{RNA}	Constant of proportionality between number of SARS-CoV 2 RNA number and amount of free viral epitopes	11.53	Estimated from [7]

Table S4. Parameter values for “Complete model”

Parameter	**Definition**	**Value**	**Ref.**
π_v*	Rate of viral particles production by pneumocytes	166625 V/(cells·day)	[3], [24]
α_c*	Rate constant for the antibody–virus complex engulfment	0.001198	Estimated from [7]
ρ*	Probability of macrophages to become infected	ρ/25	[3], [21]

Table S5. The sensitivity analysis of the "Model-Ab"

Parameters of the model	Relative sensitivity									
	AP	APS_{lung}	APS_{lo}	V_f	Va	B	Bm	Pl	A_{lung}	A_{lo}
A_{RNA}	-0.008	2.22	1.97	0.19	1.31	$1.6\cdot10^{-4}$	0.82	0.85	0.96	0.95
x_0	-0.003	0.003	-0.12	0.09	1.7	$-9\cdot10^{-6}$	0.59	0.62	0.69	0.69
w_1	-0.003	0.59	0.51	-0.25	0.69	$4\cdot10^{-5}$	0.32	0.36	0.37	0.37
w_2	0.004	0.85	0.53	0.2	-0.08	$4\cdot10^{-5}$	-0.83	-0.93	0.95	0.95
w_3	-0.008	3.34	2.93	9.9	11.35	$2\cdot10^{-4}$	0.96	1.38	1.11	1.11
β_{RNA}	-0.008	2.26	2.01	0.20	1.3	$2\cdot10^{-4}$	0.82	0.86	0.96	0.95
k_{v-1}	$3\cdot10^{-7}$	-0.003	$1\cdot10^{-4}$	-0.03	0.009	$1\cdot10^{-8}$	$-1\cdot10^{-4}$	$1\cdot10^{-4}$	$-2\cdot10^{-4}$	$-2\cdot10^{-4}$
K_d	$-1\cdot10^{-4}$	0.10	0.13	0.68	-0.26	$8\cdot10^{-6}$	0.01	0.03	0.01	0.01
C_{v1}	$-1\cdot10^{-7}$	$8\cdot10^{-5}$	$7\cdot10^{-5}$	$5\cdot10^{-4}$	$-2\cdot10^{-4}$	$5\cdot10^{-9}$	$9\cdot10^{-6}$	$2\cdot10^{-5}$	10^{-5}	10^{-5}
C_{v2}	10^{-7}	$-7\cdot10^{-5}$	$-6\cdot10^{-5}$	$-4\cdot10^{-4}$	10^{-4}	$-5\cdot10^{-9}$	$-9\cdot10^{-6}$	$-2\cdot10^{-5}$	-10^{-5}	-10^{-5}
χ_{ap}	0.03	1.05	0.75	0.02	-0.007	$6\cdot10^{-5}$	-0.73	-0.75	-0.84	-0.83
β_{apm}	-0.004	0.26	0.24	-0.08	0.03	$2\cdot10^{-5}$	0.09	0.14	0.10	0.10
ϕ	0.008	-0.56	-0.67	0.28	-0.11	$-5\cdot10^{-5}$	-0.85	-0.98	-0.97	-0.97
δ_s	$-2\cdot10^{-4}$	-1.50	-0.87	0.3	-0.11	$-6\cdot10^{-5}$	-0.99	-1.53	-1.13	-1.16
k_{s1}	$-8\cdot10^{-4}$	-0.58	0.49	-0.62	0.23	$4\cdot10^{-5}$	0.73	0.77	0.84	0.83
k_{s-1}	$-8\cdot10^{-5}$	1.06	0.94	-1.2	0.46	$7\cdot10^{-5}$	0.33	0.26	0.38	0.37
ν	$-5\cdot10^{-5}$	0.32	0.23	1.28	-0.49	-0.99	-1.39	-1.51	-0.70	-0.79
χ_b	-10^{-5}	-0.05	-0.04	-1.26	0.48	$-8\cdot10^{-5}$	0.01	-0.01	0.01	0.01
π_b	0.005	-0.13	-0.34	0.45	-0.17	$7\cdot10^{-5}$	-0.91	-1.06	1.04	-1.04
π_{diff}	$-8\cdot10^{-6}$	1.07	0.85	-1.66	0.63	$4\cdot10^{-5}$	0.92	1.02	1.05	1.05
t_{naive}	$-9\cdot10^{-4}$	1.97	1.64	1.32	-0.50	10^{-4}	-1.89	-0.95	-2.03	-1.95
δ_{pl}	$-5\cdot10^{-5}$	0.30	1.99	0.18	-0.07	$2\cdot10^{-5}$	0.002	-1.97	-0.82	-0.91
k_{bm1}	$-5\cdot10^{-10}$	$5\cdot10^{-6}$	$7\cdot10^{-7}$	$7\cdot10^{-5}$	$-3\cdot10^{-5}$	$5\cdot10^{-11}$	$-5\cdot10^{-4}$	-10^{-5}	$-5\cdot10^{-6}$	$-5\cdot10^{-6}$
k_{bm2}	$5\cdot10^{-6}$	$5\cdot10^{-6}$	$6\cdot10^{-7}$	$8\cdot10$	$-3\cdot10^{-5}$	$4\cdot10^{-11}$	$7\cdot10^{-4}$	$2\cdot10^{-5}$	$7\cdot10^{-6}$	$8\cdot10^{-6}$
α_{pl}	$-6\cdot10^{-6}$	0.86	0.73	-1.22	0.46	$6\cdot10^{-5}$	-0.02	-0.05	0.98	0.98
δ_a	-10^{-5}	0.02	0.01	0.30	-0.11	$9\cdot10^{-7}$	0.001	0.003	-0.41	-0.38
ka_1	$-3\cdot10^{-5}$	0.03	0.02	0.20	-0.08	$2\cdot10^{-6}$	0.002	0.006	-0.27	0.65
ka_{-1}	$5\cdot10^{-5}$	0.21	0.13	-0.63	0.24	10^{-5}	-0.008	-0.03	0.29	-0.72

Table S6. Donor-specific parameters for the "Model-Ab".

Experimental results were taken from [1]. Corresponding model and experimental results of antibodies response for each patient are represented on Figure SI2, SI3. Patient "G" with severe case is represented on Figure 1c.

Patient, severity	w_3, days	β_{RNA}	t_{naive}, days	ω	π_{pl} $(cells\cdot days)^{-1}$	δ_{pl}, $days^{-1}$
A, Mild case	1.76	615	6.09	1.238	$6.7\cdot10^{-5}$	0.00024
B, Mild case	3.658	429	8.44	1.0745	$3.97\cdot10^{-5}$	0.0066
C, Mild case	1.09	496	10.2362	1.096	$1.34\cdot10^{-4}$	0.002877
D, Mild case	1.5	745	11.0284	1.42	$4.06\cdot10^{-5}$	0.0019

E, Mild case	2.94	909	6.9988	1.186	$1.32 \cdot 10^{-5}$	0.006156
F, Mild case	2.4	537	11.6682	1.16505	$4.84 \cdot 10^{-5}$	0.000126
A, Severe case	1.447	226	7.4353	1.1	$2 \cdot 10^{-4}$	0.6807
B, Severe case	2.4768	243	14.5918	1.028	$1.32 \cdot 10^{-4}$	0.7149
C, Severe case	1.59	595	13.7439	1.002	$1.45 \cdot 10^{-4}$	0.004638
D, Severe case	1.64	668	10.3878	1.0807	$1.25 \cdot 10^{-4}$	0.002757
E, Severe case	3.7482	423	11.1476	1.11718	$1.86 \cdot 10^{-4}$	0.278
F, Severe case	3.99	772	11.9372	1.50957	$1.55 \cdot 10^{-4}$	0.4527
G, Severe case	2.62	264	12.8159	1.04	$3.47 \cdot 10^{-5}$	0.004284

Table S7. Donor-specific parameters for infection propagation module ("Model-Cv").

Experimental results were taken from [7]. Corresponding model and experimental results of viral load dynamic for each patient are represented on Figure SI3. Patient "G" is represented on Figure 1d.

Patient	β_{inf}, (Virus·days)$^{-1}$	δ_v. days^{-1}	a_{c0}, (cells^{2}days)$^{-1}$	Virus number (initial values)
Patient A	$1.94 \cdot 10^{-6}$	122	$1.28 \cdot 10^{-10}$	$1.31 \cdot 10^{7}$
Patient B	$3.44 \cdot 10^{-6}$	228	$1.3 \cdot 10^{-12}$	$4.94 \cdot 10^{7}$
Patient C	$4.31 \cdot 10^{-6}$	225	$2.64 \cdot 10^{-10}$	$5 \cdot 10^{7}$
Patient D	$1.35 \cdot 10^{-5}$	234	$8.03 \cdot 10^{-12}$	$4.97 \cdot 10^{7}$
Patient E	$3.55 \cdot 10^{-6}$	159	$3.5 \cdot 10^{-9}$	$1.01 \cdot 10^{5}$
Patient F	$4.05 \cdot 10^{-6}$	230	$8.01 \cdot 10^{-12}$	$1.16 \cdot 10^{7}$
Patient G	$2.21 \cdot 10^{-6}$	255	$2.17 \cdot 10^{-10}$	$1.32 \cdot 10^{7}$

Table S8. **Parameter values for supplemented equations**

Parameter	Definition	Value	Ref.
δ^*_v	Thermal degradation of viral particles	10 day^{-1}	[26]
β^*_{inf}	Rate of HAE cells infection	$1.4 \cdot 10^{-10}$ (Virus·day)$^{-1}$	[24]
k_{v1old}	Virus-antibody association rate	$k_{v-1}/(K_d \cdot 6 \cdot 10^{20}) = 4.3 \cdot 10^{-12}$ V·days$^{-1.}$	[4], [5]
k_{v-1old}	Virus-antibody dissociation rate	25.8 days^{-1}	[4], [5]
K_{dold}	Virus-antibody dissociation constant	$1 \cdot 10^{-8}$ M	[4]

2. Supporting Figures

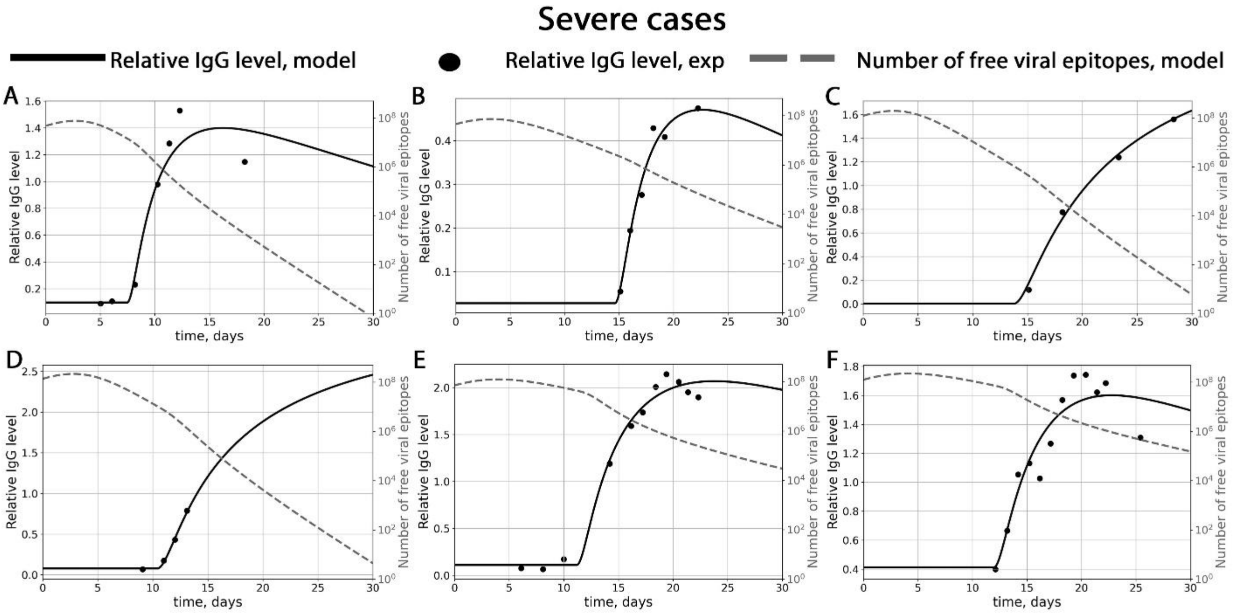

Figure S1. Best fit of the antibody-producing module (“Model-Ab”) to the antibodies response in patient with mild cases of SARS-CoV 2 infection [1]. Experimental results of relative IgG level in patients are represented by points; results of relative IgG level are represented by black solid lines; number of free viral epitopes for each patient are represented by grey dashed lines. Donor-specific parameters: (A) ω=1.24, t_{naive}=6.09 days, δ_{pl}=2.4·10^{-4} $days^{-1}$, π_{pl}=6.7·10^{-5}(cells ·days)$^{-1}$, β_{RNA}=615, w_3=1.76 days; (B) ω=1.07, t_{naive}=8.44 days, δ_{pl}=0.007 $days^{-1}$, π_{pl}=4·10^{-5} (cells ·days)$^{-1}$, β_{RNA}=429, w_3=3.37 days; (C) ω=1.09, t_{naive}=10.23 days, δ_{pl}=0.003 $days^{-1}$, π_{pl}=1.3·10^{-4} (cells ·days)$^{-1}$, β_{RNA}=496, w_3=1.09 days; (D) ω=1.42, t_{naive}=11.03 days, δ_{pl}=0.002 $days^{-1}$, π_{pl}=4.06·10^{-5} (cells·days)$^{-1}$, β_{RNA}=745, w_3=1.51 days; (E) ω=1.32, t_{naive}=6.99 days, δ_{pl}=0.006 $days^{-1}$, π_{pl}=1.32·10^{-5} (APS·days)$^{-1}$, β_{RNA}=908, w_3=2.94 days; (F) ω=1.16, t_{naive}=11.67 days, δ_{pl}=1.2·10^{-4} $days^{-1}$, π_{pl}=4.8·10^{-5} (cells ·days)$^{-1}$, β_{RNA}=536, w_3=1.16 days.

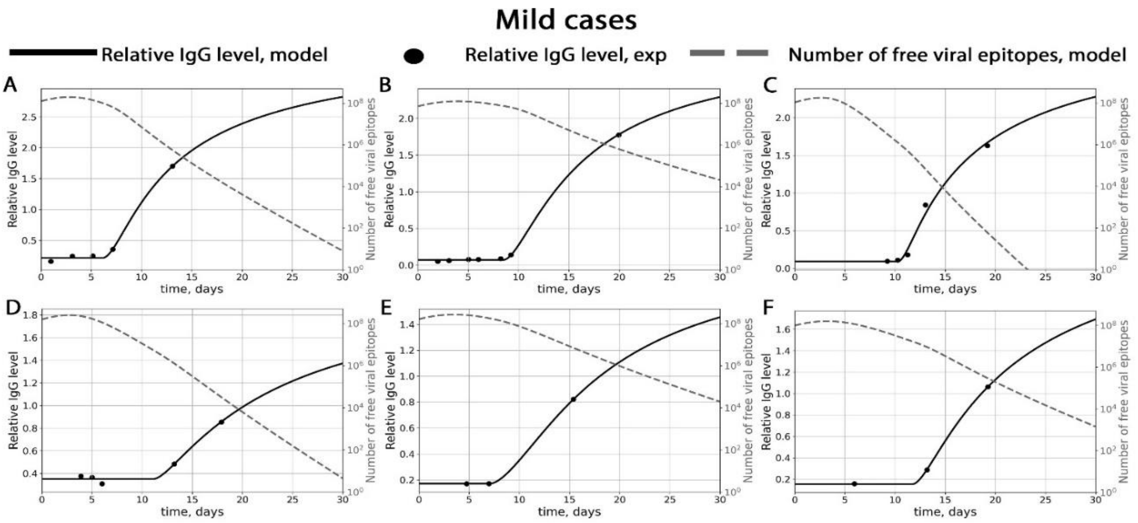

Figure S2. **Best fit of the antibody-producing module (“Model-Ab”) to the antibodies response in patient with severe cases of SARS-CoV 2 infection** [1]. Experimental results of relative IgG level in patients are represented by points; results of relative IgG level are represented by black solid lines; number of free viral epitopes for each patient are represented by grey dashed lines. Donor-specific parameters: (A) ω=1.1, t_{naive}=7.44 days, δ_{pl}=0.68·10^{-4} $days^{-1}$, π_{pl}=2·10^{-4} (APS·days)$^{-1}$, β_{RNA}=226, w_3=1.45 days; (B) ω=1.03, t_{naive}=14.6 days, δ_{pl}=0.71 $days^{-1}$, π_{pl}=1.3·10^{-4} (APS·days)$^{-1}$, β_{RNA}=243, w_3=2.48 days; (C) ω=1, t_{naive}=13.74 days, δ_{pl}=0.006 $days^{-1}$, π_{pl}=1.4·10^{-4} (APS·days)$^{-1}$, β_{RNA}=596, w_3=1.59 days; (D) ω=1.08, t_{naive}=10.39 days, δ_{pl}=0.003 $days^{-1}$, π_{pl}=1.2·10^{-4} (APS·days)$^{-1}$, β_{RNA}=668, w_3=1.64 days; (E) ω=1.12, t_{naive}=11.15 days, δ_{pl}=0.278 $days^{-1}$, π_{pl}=1.8·10^{-4} (APS·days)$^{-1}$, β_{RNA}=423, w_3=3.74 days; (F) ω=1.5, t_{naive}=11.9 days, δ_{pl}=0.45 $days^{-1}$, π_{pl}=1.5·10^{-4} (cells·days)$^{-1}$, β_{RNA}=772, w_3=3.99 days.

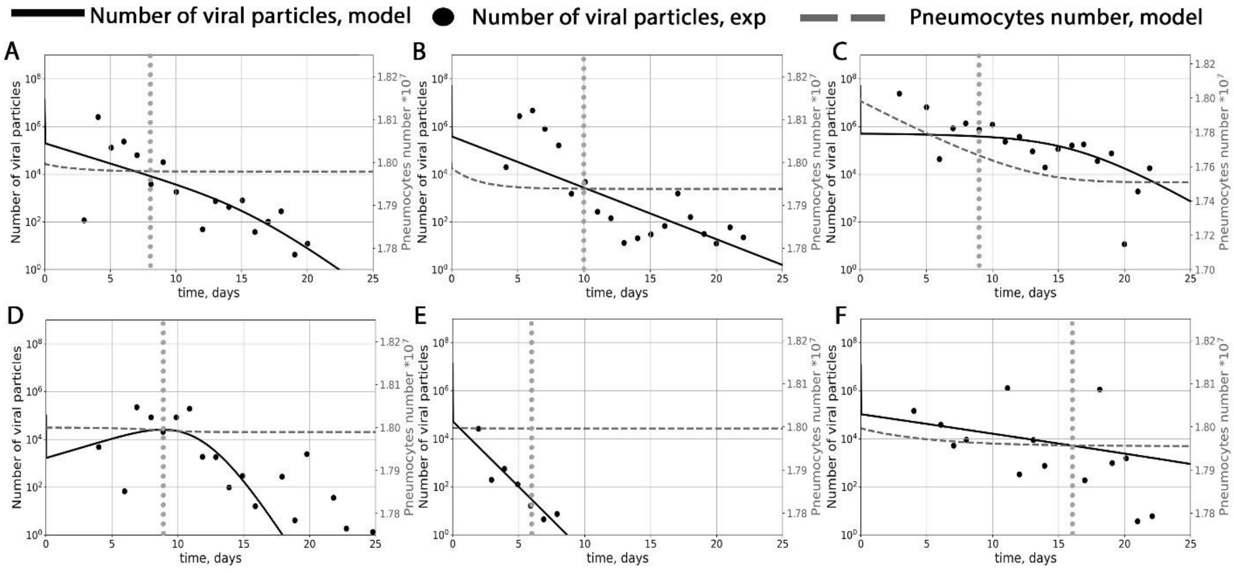

Figure S3. Best fit of the infection propagation module ("Model-Cv") to RNA-dynamics in patients with SARS-CoV 2 infecsion [7]. Experimental results of viral load in patients are represented by points; model viral dynamics are represented by black solid lines, pneumocyte kinetics are marked by grey dashed lines. Days of seroconversion are indicated by vertical grey dotted lines. Donor-specific parameters: (A) β_{inf} =1.94·10^{-6} (Virus·days)$^{-1}$, δ_v=122 days^{-1}, ac$_0$=1.28·10^{-10}, Virus$_0$=1.31·10^7; (B) β_{inf} =3.44·10^{-6} (Virus·days)$^{-1}$, δ_v=228 days^{-1}, ac$_0$=1.3·10^{-12}, Virus$_0$=4.94·10^7; (C) β_{inf} =4.31·10^{-6} (Virus·days)$^{-1}$, δ_v=225 days^{-1}, ac$_0$=2.64·10^{-10}, Virus$_0$=5·10^7; (D) β_{inf} =1.35·10^{-6} (Virus·days)$^{-1}$, δ_v=234 days^{-1}, ac$_0$=8.03·10^{-12}, Virus$_0$=4.97·10^7; (E) β_{inf} =3.55·10^{-6} (Virus·days)$^{-1}$, δ_v=159 days^{-1}, ac$_0$=1.5·10^{-9}, Virus$_0$=1.01·10^5; (F) β_{inf} =4.05·10^{-6} (Virus·days)$^{-1}$, δ_v=230 days^{-1}, ac$_0$=8.01·10^{-12}, Virus$_0$=1.16·10^7.

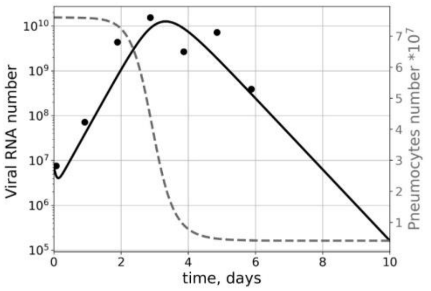

Figure S4. Best fit of simplified model to SARS-CoV 2 RNA dynamic in culture of HAE cells [24]. Experimental results of viral load in HAE cells are represented by points; model viral dynamic is represented by black solid line, pneumocyte kinetic is marked by grey dashed line.

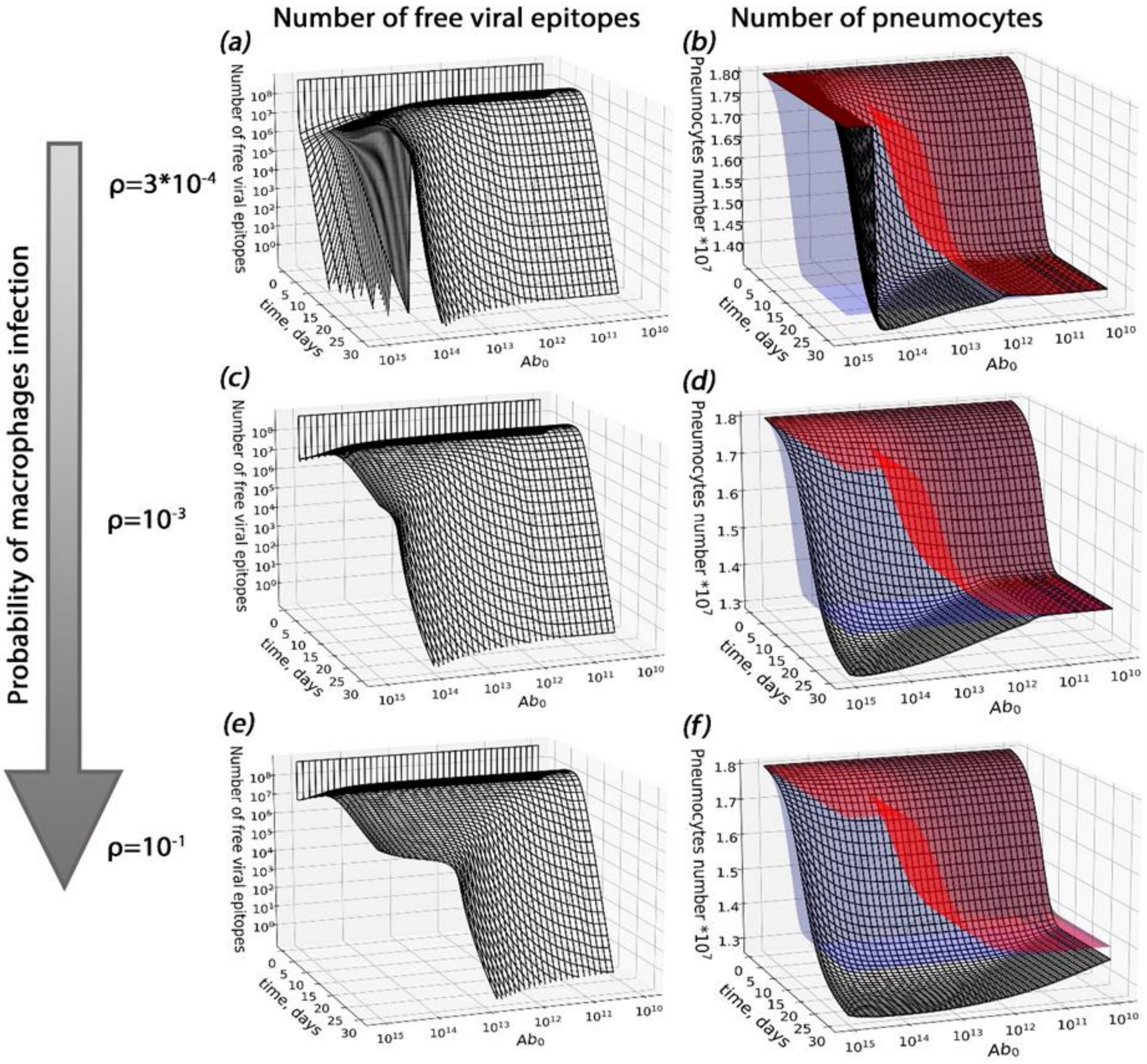

Figure S5. Influence of pre-existing antibody concentration on the viral load dynamics and pneumocyte survival at different macrophage infection probability (ρ). Viral dynamics *(a)* and pneumocyte amount kinetics *(b)* in presence of different pre-existing antibody concentration at intermediate macrophage infection probability ($\rho = 3\cdot10^{-4}$). Viral dynamics (*c*) and pneumocyte amount kinetics (*d*) in presence of different pre-existing antibody concentration at moderate macrophage infection probability ($\rho = 10^{-3}$). Viral dynamics (*e*) and pneumocyte amount kinetics (*f*) in presence of different pre-existing antibody concentration at high macrophage infection probability ($\rho = 0.1$). Red surface denotes pneumocyte amount kinetics in the absence of macrophage infection (ρ_{old}=0), constant K_{dold} (K_{dold} = 10 nM) in presence of different pre-existing antibody concentration. Blue surface denotes pneumocyte amount kinetics in absence of macrophage infection (ρ_{old} = 0) and pre-existing antibody concentration $Ab_o = 0$.

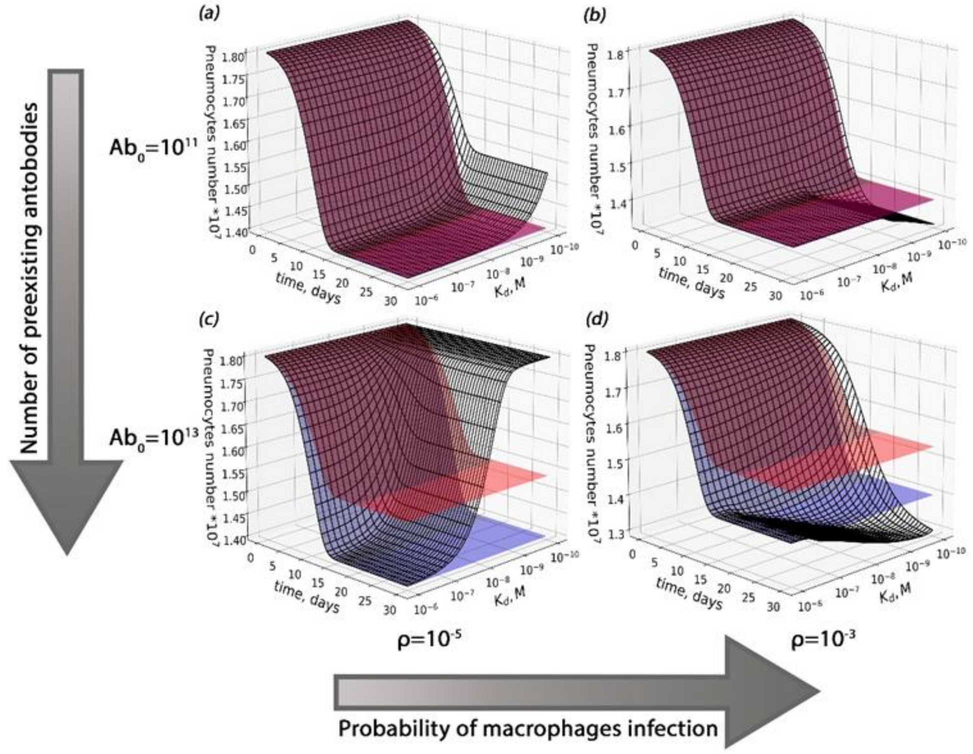

Figure S6. Influence of pre-existing antibodies at different macrophages infection probability on possible ADE occurrence. Dependence of pneumocyte numbers on time and K_d values for various macrophage infection probabilities, low ($\rho = 10^{-5}$, *(a)*,*(c)*) and moderate ($\rho = 10^{-3}$, *(b)*,*(d)*) and various concentrations of pre-existing antibodies, intermediate ($Ab_{old}=10^{11}$, *(a-b)*) and high ($Ab_{old}=10^{13}$, *(c-d)*). Red surface denotes pneumocyte numbers in absence of macrophage infection and K_{dold} = 10 nM. Blue surface denotes pneumocyte numbers in absence of macrophage infection and pre-existing antibodies.

3. Supporting Methods for model description and parameters estimation

S1. Parameters for antibody-response generation module (“Model-Ab”)

The parameters for the naïve B-cells proliferation, parameters for short-lived plasma cells differentiation, parameters of the antibody secretion and death rates were taken from published mathematical models and experimental data [14], [20], [27], [28]. The relationship between the number of long-lived plasma cells and memory B-cells depends on several parameters, including antigen affinity, strength of T-cells help, activity of such transcriptional factors as IRF4 and Bach2 and other circumstances [29]. In the model we assumed that B-lymphocytes have equal probability to differentiate into plasma or memory B-cells.

Although there could be different antibodies in a given organism with different affinities to the same antigen, or with affinities to different antigens, in the “Model-Ab” we assumed that all antibodies had the same average affinity to the antigens on the viral particle [5].

Process of memory B-cells differentiation into plasma cells has been described by parameters t_{memory} and π_{ml}. These parameters were adjusted for the model to be in accord with the following reasoning. The differentiation rate from memory B-cells into plasma cells should be small and does not have much influence at first infection, but have to be high enough to provide early appearance of plasma cells and antibodies upon reinfection. All chosen parameters are represented in Tables S1, S2.

S2. Parameters for “Complete model”

First of all, based on experimental data about SARS-CoV-2 replication in human airway epithelial (HAE) cells [24], we estimated the parameters viral replication rate (π_V^*) and infected pneumocyte death rate (δ_p). We took into account that the experiment was carried out on cell culture, where there are no immune cells, virus clearance, or possible interaction with antibodies, so the initial numbers of naïve and memory B-cells, plasma cells and macrophages were set to zero. Moreover, it was supposed that no more 90% of cells die during viral infection. The correspondence between experimental data and model results are represented on [24]. Antibody concentration dynamics and seroconversion times in the united model were qualitatively similar to the results from antibody generation module.

As it was estimated in previous works, macrophages phagocytosis rate lies in range 10^{-7} to 10^{-5} units per second (or 0.01 to 1 units per day) [20], [25]. To determine appropriate parameter values of the model we fixed macrophages engulfment rate of viral particles (α_v) and varied initial macrophages number to fit the data on viral RNA concentration in the sputum of individual patients with confirmed SARS-CoV-2 [7]. It is important that the rate of viral removal by immune system should be higher than the natural degradation of the virus as a result of thermal fluctuations. We also took into account this fact choosing the appropriate parameters.

S3. Reduced equation system of viral propagation in culture of HAE cells

$$\frac{dVirus}{dt} = pP_i - {\delta^*}_v * Virus, \quad \text{(SI1)}$$

$$\frac{dP_n}{dt} = -{\beta^*}_{inf} P_n * Virus, \quad \text{(SI2)}$$

$$\frac{dP_i}{dt} = {\beta^*}_{inf} P_n * Virus - \delta_p P_i, \quad \text{(SI3)}$$

where ${\delta^*}_v$ is thermal degradation of viral particles, ${\beta^*}_{inf}$ is the rate of HAE cells infection. Additional parameters values are represented in Table S8.

S4. Modified system of equations, including interaction with pre-existing antibodies

$$\frac{dV}{dt} = \pi_V^* P_i - k_{v1} V * Ab_{lung} + k_{v-1} Va - \left(\alpha_v \; * M_A + \frac{C_{v1}}{C_{v2}+V}\right) V - k_{v1\_old} V * Ab_{lung\_old} + k_{v-1\_old} Va_{old}, \quad \text{(SI4)}$$

$$\frac{dVa_{old}}{dt} = k_{v1\_old} V * Ab_{lung\_old} - k_{v-1\_old} Va_{old} - \alpha_c^* * M_A * Va_{old}, \quad \text{(SI5)}$$

$$\frac{dM_A}{dt} = \chi_M (M_0 - M_A) - \rho_{old}^* \alpha_c^* \; Va_{old} M_A + \sigma M^*, \quad \text{(SI6)}$$

$$\frac{dM^*}{dt} = \rho^*_{old}\alpha^*_c V a_{old} * M_A - (\sigma + \delta_I)M^*, \quad \text{(SI7)}$$

$$\frac{dAb_{lung\_old}}{dt} = -k_{v1\_old}V * Ab_{lung\_old} + k_{v-1\_old}Va_{old} + \nu(ka_{-1} * Ab_{lo\_old} - ka_1 Ab_{lung\_old}) - \delta_A Ab_{lung\_old}, \quad \text{(SI8)}$$

$$\frac{dAb_{lo\_old}}{dt} = ka_{-1} * Ab_{lo\_old} + ka_1 Ab_{lung\_old} - \delta_A Ab_{lo\_old}, \quad \text{(SI9)}$$

$\rho*_{old}$ equals ρ_{old} divided to number of S proteins on viral particle surface [3]. Additional parameters values are represented in Table S8.

4. Supporting Abbreviations

ACE 2 - Angiotensin-converting enzyme 2

ADE - Antibody-dependent enhancement

APS - antigen-presenting site

Bm – memory B-cell

COVID-19 - COronaVirus Infectious Disease-2019

HIV - Human immunodeficiency viruses

PCR - polymerase chain reaction

Pl - plasma cell

5. Supporting References

[1] K. K.-W. To *et al.*, 'Temporal profiles of viral load in posterior oropharyngeal saliva samples and serum antibody responses during infection by SARS-CoV-2: an observational cohort study', *Lancet Infect. Dis.*, vol. 20, no. 5, pp. 565–574, May 2020, doi: 10.1016/S1473-3099(20)30196-1.

[2] R. Sender *et al.*, 'The total number and mass of SARS-CoV-2 virions', *Proc. Natl. Acad. Sci.*, vol. 118, no. 25, Jun. 2021, doi: 10.1073/pnas.2024815118.

[3] Z. Ke *et al.*, 'Structures and distributions of SARS-CoV-2 spike proteins on intact virions', *Nature*, vol. 588, no. 7838, pp. 498–502, Dec. 2020, doi: 10.1038/s41586-020-2665-2.

[4] X. Chi *et al.*, 'Humanized single domain antibodies neutralize SARS-CoV-2 by targeting the spike receptor binding domain', *Nat. Commun.*, vol. 11, no. 1, Art. no. 1, Sep. 2020, doi: 10.1038/s41467-020-18387-8.

[5] E. Seydoux *et al.*, 'Characterization of neutralizing antibodies from a SARS-CoV-2 infected individual', *bioRxiv*, May 2020, doi: 10.1101/2020.05.12.091298.

[6] R. F. Reis *et al.*, 'A Validated Mathematical Model of the Cytokine Release Syndrome in Severe COVID-19', *Front. Mol. Biosci.*, vol. 8, p. 680, 2021, doi: 10.3389/fmolb.2021.639423.

[7] R. Wölfel *et al.*, 'Virological assessment of hospitalized patients with COVID-2019', *Nature*, vol. 581, no. 7809, Art. no. 7809, May 2020, doi: 10.1038/s41586-020-2196-x.

[8] A. T. Kamath, S. Henri, F. Battye, D. F. Tough, and K. Shortman, 'Developmental kinetics and lifespan of dendritic cells in mouse lymphoid organs', *Blood*, vol. 100, no. 5, pp. 1734–1741, Sep. 2002, doi: 10.1182/blood.V100.5.1734.h81702001734_1734_1741.

[9] P. Sathe and K. Shortman, 'The steady-state development of splenic dendritic cells', *Mucosal Immunol.*, vol. 1, no. 6, pp. 425–431, Nov. 2008, doi: 10.1038/mi.2008.56.

[10] K. Liu, C. Waskow, X. Liu, K. Yao, J. Hoh, and M. Nussenzweig, 'Origin of dendritic cells in peripheral lymphoid organs of mice', *Nat. Immunol.*, vol. 8, no. 6, pp. 578–583, Jun. 2007, doi: 10.1038/ni1462.

[11] G. E. Hartley *et al.*, 'Rapid generation of durable B cell memory to SARS-CoV-2 spike and nucleocapsid proteins in COVID-19 and convalescence', *Sci. Immunol.*, vol. 5, no. 54, p. eabf8891, Dec. 2020, doi: 10.1126/sciimmunol.abf8891.

[12] M. Mamani-Matsuda *et al.*, 'The human spleen is a major reservoir for long-lived vaccinia virus–specific memory B cells', *Blood*, vol. 111, no. 9, pp. 4653–4659, May 2008, doi: 10.1182/blood-2007-11-123844.

[13] I. Bujoreanu and V. Gupta, 'Anatomy, Lymph Nodes', in *StatPearls*, Treasure Island (FL): StatPearls Publishing, 2022. Accessed: Jul. 18, 2022. [Online]. Available: http://www.ncbi.nlm.nih.gov/books/NBK557717/

[14] C. R. B. Bonin, G. C. Fernandes, R. W. dos Santos, and M. Lobosco, 'A qualitatively validated mathematical-computational model of the immune response to the yellow fever vaccine', *BMC Immunol.*, vol. 19, no. 1, p. 15, Dec. 2018, doi: 10.1186/s12865-018-0252-1.

[15] I. J. Amanna and M. K. Slifka, 'Mechanisms that determine plasma cell lifespan and the duration of humoral immunity', *Immunol. Rev.*, vol. 236, no. 1, pp. 125–138, Jul. 2010, doi: 10.1111/j.1600-065X.2010.00912.x.

[16] R. Itoua Maïga, G. Bonnaure, J. Tremblay Rochette, and S. Néron, 'Human CD38hiCD138+ Plasma Cells Can Be Generated In Vitro from CD40-Activated Switched-Memory B Lymphocytes', *J. Immunol. Res.*, vol. 2014, p. 635108, 2014, doi: 10.1155/2014/635108.

[17] B. Alberts, A. Johnson, J. Lewis, M. Raff, K. Roberts, and P. Walter, 'B Cells and Antibodies', *Mol. Biol. Cell 4th Ed.*, 2002, Accessed: Jul. 19, 2022. [Online]. Available: https://www.ncbi.nlm.nih.gov/books/NBK26884/

[18] T. Hibi and H. M. Dosch, 'Limiting dilution analysis of the B cell compartment in human bone marrow', *Eur. J. Immunol.*, vol. 16, no. 2, pp. 139–145, Feb. 1986, doi: 10.1002/eji.1830160206.

[19] M. Arvola *et al.*, 'Immunoglobulin-Secreting Cells of Maternal Origin Can Be Detected in B Cell-Deficient Mice1', *Biol. Reprod.*, vol. 63, no. 6, pp. 1817–1824, Dec. 2000, doi: 10.1095/biolreprod63.6.1817.

[20] H. Y. Lee *et al.*, 'Simulation and Prediction of the Adaptive Immune Response to Influenza A Virus Infection', *J. Virol.*, vol. 83, no. 14, pp. 7151–7165, Jul. 2009, doi: 10.1128/JVI.00098-09.

[21] M. Cerón Gómez and H. M. Yang, 'A simple mathematical model to describe antibody-dependent enhancement in heterologous secondary infection in dengue', *Math. Med. Biol. J. IMA*, vol. 36, no. 4, pp. 411–438, Dec. 2019, doi: 10.1093/imammb/dqy016.

[22] K. A. Pawelek, G. T. Huynh, M. Quinlivan, A. Cullinane, L. Rong, and A. S. Perelson, 'Modeling Within-Host Dynamics of Influenza Virus Infection Including Immune Responses', *PLoS Comput. Biol.*, vol. 8, no. 6, p. e1002588, Jun. 2012, doi: 10.1371/journal.pcbi.1002588.

[23] S. Wang, Y. Pan, Q. Wang, H. Miao, A. N. Brown, and L. Rong, 'Modeling the viral dynamics of SARS-CoV-2 infection', *Math. Biosci.*, vol. 328, p. 108438, Oct. 2020, doi: 10.1016/j.mbs.2020.108438.

[24] N. Zhu *et al.*, 'Morphogenesis and cytopathic effect of SARS-CoV-2 infection in human airway epithelial cells', *Nat. Commun.*, vol. 11, no. 1, Art. no. 1, Aug. 2020, doi: 10.1038/s41467-020-17796-z.

[25] T. S. Kapellos *et al.*, 'A novel real time imaging platform to quantify macrophage phagocytosis', *Biochem. Pharmacol.*, vol. 116, pp. 107–119, Sep. 2016, doi: 10.1016/j.bcp.2016.07.011.

[26] T. F. Yap, Z. Liu, R. A. Shveda, and D. J. Preston, 'A predictive model of the temperature-dependent inactivation of coronaviruses', *Appl. Phys. Lett.*, vol. 117, no. 6, p. 060601, Aug. 2020, doi: 10.1063/5.0020782.

[27] E. Bromage, R. Stephens, and L. Hassoun, 'The third dimension of ELISPOTs: quantifying antibody secretion from individual plasma cells', *J. Immunol. Methods*, vol. 346, no. 1–2, pp. 75–79, Jul. 2009, doi: 10.1016/j.jim.2009.05.005.

[28] D. C. Nguyen, C. J. Joyner, I. Sanz, and F. E.-H. Lee, 'Factors Affecting Early Antibody Secreting Cell Maturation Into Long-Lived Plasma Cells', *Front. Immunol.*, vol. 10, p. 2138, 2019, doi: 10.3389/fimmu.2019.02138.

[29] B. J. Laidlaw and J. G. Cyster, 'Transcriptional regulation of memory B cell differentiation', *Nat. Rev. Immunol.*, pp. 1–12, Oct. 2020, doi: 10.1038/s41577-020-00446-2.
